# Suicide and Self-Harm Among Immigrant Youth to Ontario, Canada From Muslim Majority Countries: A Population-Based Study

**DOI:** 10.1177/07067437231166840

**Published:** 2023-04-10

**Authors:** Natasha Saunders, Rachel Strauss, Sarah Swayze, Alex Kopp, Paul Kurdyak, Zainab Furqan, Arfeen Malick, Muhammad Ishrat Husain, Mark Sinyor, Juveria Zaheer

**Affiliations:** 1 7979 The Hospital for Sick Children, Toronto, Canada; 2 Department of Pediatrics, 7938 University of Toronto, Toronto, Canada; 3 ICES, Toronto, Canada; 4 50010 Child Health Evaluative Sciences, SickKids Research Institute, Toronto, Canada; 5 Institute of Health Policy, Management and Evaluation, The University of Toronto, Toronto, Canada; 6 Edwin S.H. Leong Centre for Healthy Children, 7938 University of Toronto, Toronto, Canada; 7 7978 Centre for Addiction and Mental Health, Toronto, Canada; 8 Department of Psychiatry, 7938 University of Toronto, Toronto, Canada; 9 Department of Psychiatry, Sunnybrook Health Sciences Centre, Toronto, Canada; 10 University Health Network, Toronto, Canada

**Keywords:** self-harm, suicide, immigrant mental health, youth, adolescence, child and adolescent psychiatry, Muslim, religion, surveillance, cohort study

## Abstract

**Objective:**

To examine the association between Muslim religious affiliation and suicide and self-harm presentations among first- and second-generation immigrant youth.

**Methods:**

We performed a population-based cohort study involving individuals aged 12 to 24 years, living in Ontario, who immigrated to Canada between 1 January 2003 and 31 May 2017 (first generation) and those born to immigrant mothers (second generation). Health administrative and demographic data were used to analyze suicide and self-harm presentations. Sex-stratified logistic regression models generated odds ratios (OR) for suicide and negative binomial regression models generated rate ratios (aRR) for self-harm presentations, adjusting for refugee status and time since migration.

**Results:**

Of 1,070,248 immigrant youth (50.1% female), there were 129,919 (23.8%) females and 129,446 (24.2%) males from Muslim-majority countries. Males from Muslim-majority countries had lower suicide rates (3.8/100,000 person years [PY]) compared to males from Muslim-minority countries (5.9/100,000 PY) (OR: 0.62, 95% CI, 0.42–0.92). Rates of suicide between female Muslim-majority and Muslim-minority groups were not different (Muslim-majority 1.8/100,000 PY; Muslim-minority 2.2/100,000 PY) (OR: 0.82, 95% CI, 0.46–1.47). Males from Muslim-majority countries had lower rates of self-harm presentations than males from Muslim-minority (<10%) countries (Muslim majority: 12.2/10,000 PY, Muslim-minority: 14.1/10,000 PY) (aRR: 0.82, 95% CI, 0.75, 0.90). Among female immigrants, rates of self-harm presentations were not different among Muslim-majority (30.1/10,000 PY) compared to Muslim-minority (<10%) (32.9/10,000 PY) (aRR: 0.93, 95% CI, 0.87–1.00) countries. For females, older age at immigration conferred a lower risk of self-harm presentations.

**Conclusion:**

Being a male from a Muslim-majority country may confer protection from suicide and self-harm presentations but the same was not observed for females. Approaches to understanding the observed sex-based differences are warranted.

## Résumé

**Objectif:**

Examiner l'association entre l'affiliation religieuse musulmane et les présentations sur le suicide et l'autodestruction chez les jeunes immigrants de la première et de la deuxième génération.

**Méthodes:**

Nous avons mené une étude de cohorte dans la population impliquant des personnes âgées de 12 à 24 ans, habitant en Ontario, qui ont immigré au Canada entre le 1er janvier 2003 et le 31 mai 2017 (première génération) et celles nées de mère immigrante (deuxième génération). Les données de santé administratives et démographiques ont servi à analyser les présentations sur le suicide et l'autodestruction. Des modèles de régression logistique stratifiée par sexe ont généré des rapports de cotes (RC) pour le suicide et des modèles de régression binomiale négative ont généré des ratios de taux (RTa) pour les présentations sur l'autodestruction, en ajustant pour le statut de réfugié et le temps depuis la migration.

**Résultats:**

Sur 1070,248 jeunes immigrants, (50,1% des femmes), il y avait 129919 (23,8%) des femmes et 129446 (24,2%) des hommes de pays en majorité musulmans. Les hommes des pays en majorité musulmans avaient des taux de suicide plus faibles (3,8/100000 années-personnes [AP]) comparé aux hommes des pays en minorité musulmans (5,9/100000 AP) (RC : 0,62, IC à 95%, 0,42 à 0,92). Les taux de suicide entre les femmes de majorité musulmane et les groupes de minorité musulmane n'étaient pas différents (majorité musulmane 1,8/100000 AP; minorité musulmane 2,2/100000 AP) (RC : 0,82, IC à 95%, 0,46 à 1,47). Les hommes des pays en majorité musulmans avaient des taux plus faibles de présentations sur l'autodestruction que les hommes des pays en minorité musulmans (< 10%). (Majorité musulmane : 12,2/10000 AP, minorité musulmane : 14,1/10000 AP) (RTa : 0,82, IC à 95%, 0,75 à 0,90). Chez les femmes immigrantes, les taux des présentations sur autodestruction n'étaient pas différents chez les pays à la majorité musulmane (30,1/10000 AP) comparée aux pays à la minorité musulmane (< 10%) (32,9/10000 AP) (RTa : 0,93, IC à 95%, 0,87 à 1,00). Pour les femmes, être plus âgées lors de ‘immigration comportait un risque plus faible de présentations sur l'autodestruction.

**Conclusion:**

Être un homme d'un pays majoritairement musulman peut offrir une protection contre les présentations sur le suicide et l'autodestruction, mais ce résultat n'a pas été observé chez les femmes. Les approches pour comprendre les différences observées selon le sexe sont justifiées.

## Introduction

Suicide is the second leading cause of death in 10 to 29-year-olds.^1^ Across the globe, self-harm, including self-inflicted injury or poisoning with or without the intent to die or with undetermined intent, are common reasons for emergency department visits and hospitalizations.^2^ Despite growing global interest in the epidemiology of mental health in children and youth, little is known about suicide and self-harm risk within subpopulations of youth, including immigrants and ethnic or religious minorities, and how this risk manifests across generations.^3^ Emerging evidence suggests that rates of suicide and self-harm requiring medical or psychiatric care are lower in immigrants than non-immigrants, however, large variation by country of origin and visa class is observed.^2,4^ Disentangling specific suicide risk factors, including religious or cultural affiliation, among heterogeneous immigrant populations is important to ensure adequate screening and prevention strategies and supports.

While religion and religiosity have long been identified as protective factors for suicide and suicidal behaviour,^5,6^ high-quality population-level analyses have not been conducted, including analyses that consider sex, generation, and refugee-status. Mainstream interpretations of Islamic text consider suicide to be a prohibited act. These interpretations, combined with the pro-social effects of religious affiliation, have been cited to explain low reported rates of suicide and suicidal behaviour in the Muslim world.^7^ However, because of the religious and legal prohibition against suicide in Muslim-majority countries, many deaths may not be reported or are misclassified, underestimating the true burden of suicide.^8^ Stigma towards suicidal behaviour and mental illness, along with lack of spiritually and culturally adapted mental health care for Muslim communities may also contribute to low frequency of helpseeking.^9,10^

Canada is a highly diverse country with over one million Muslims, comprising 3.2% of the population and 73% of Canadian Muslims live in Ontario.^11^ Canadian Muslims face high rates of Islamophobia^12^ and religious discrimination has been associated with lower rates of accessing health care. Adolescence and young adulthood is the time of onset of most major mental illnesses; about 20% of all Ontario youth will experience mental illness requiring access to high-quality mental health care; adolescents and young adults have the highest rate of non-fatal self-harm of any group.^13^ Young Muslims in Canada are a particularly vulnerable group, due to low rates of and difficulty accessing mental health care,^10^ demonstrated high rates of self-harm requiring emergency care in immigrant adults from several Muslim-majority countries,^4^ negotiating identity, familial, social and gender role expectations across cultures and generations, and experiences of discrimination and Islamophobia.^14^ Further, there may be sex- and/or gender-based differences, including higher rates of Islamophobia and Islamophobic violence towards visibly Muslim females,^15^ in these experiences that ultimately affect suicide and self-harm risk.

As part of our larger mixed-methods body of work to understand the role of Muslim affiliation and suicide and self-harm risk, our main objective of this quantitative arm was to test the association between Muslim religious affiliation and suicide and self-harm presentation to acute care. As an ecological (rather than individual level) measure of Muslim religious affiliation,^15^ we used the proportion of Muslim population in the country of birth (first generation) or mother's country of birth facilitated through maternal-child data linkage (second generation) of male and female first- and second-generation immigrant youth residing in Ontario, Canada. Though not all immigrants from Muslim-majority countries identify as Muslim, country-level reports of attitudes towards suicide (e.g., justifiability), subjective religiosity (e.g., the value of religion), religious practices (e.g., praying frequency, attendance at religious services) and religion denomination (e.g., Muslim, Hindu) have been shown to be associated with country-level suicide rates.^16^ We hypothesize that risks of death by suicide and self-harm are lower in immigrant youth from Muslim majority countries compared to those from countries with a minority (<10%) proportion of Muslims. We also expect that the extent of these associations will be greater among males than females.

## Methods

### Study Population and Design

We conducted a population-based cohort study of first- and second-generation immigrant youth in Ontario, Canada. To create the cohort, we included individuals 12 to 24 years old, living in Ontario, who immigrated to Canada (i.e., they were born outside Canada, first generation immigrants) between 1 January 2003 to 31 May 2017. We then included individuals 12 to 24 years old, living in Ontario, who were born in Canada to immigrant mothers (second generation immigrants). Individuals missing a birth date, sex or having an invalid health card number required for database linkages were excluded (eFigure 1). Individuals were followed for study outcomes from their arrival in Ontario until their first self-harm event, death, loss of provincial health insurance eligibility, or end of study.

### Data Sources

Health administrative and demographic data were accessed through linked datasets using unique encoded identifiers and analyzed at ICES (formerly the Institute for Clinical Evaluative Sciences) (eTable 1). ICES is an independent, non-profit research institute whose legal status under Ontario's health information privacy law allows it to collect and analyze health care and demographic data, without consent, for health system evaluation and improvement. To identify immigrants, we used Immigration, Refugees and Citizenship Canada (IRCC) Permanent Resident Database. This includes data on country of origin, visa class (e.g., refugee, family class, economic class immigrants), date of arrival in Canada, and languages spoken. Linkage to Ontario's health registry is >86%.^17^ The Canadian Institute for Health Information Discharge Abstract Database (CIHI-DAD) was used for diagnostic and procedural information on all hospitalizations and the National Ambulatory Care Reporting System (NACRS) was used for diagnostic information for emergency room visits. The Ontario Registrar General Vital Statistics—Deaths (ORGD) registry was used to ascertain suicides. The ICES-derived MOMBABY database was used to identify mother-infant dyads for individuals born in Ontario hospitals. This allowed for identification of youth born to immigrant mothers (second generation immigrants). There is currently no linkage available to ascertain father-infant dyads in existing datasets. Sociodemographic information including age, sex, and postal code were obtained from the Registered Persons Database (RPDB), Ontario's health care registry and linked to Statistics Canada's Postal Code Conversion File to obtain area-based information including neighborhood-level income and rurality. The Ontario Marginalization Index (ON-MARG), a census- and geographically-based index, was used to determine neighbourhood level material deprivation (i.e., inability to access and attain basic material needs).

### Exposures

Our primary exposure variable, Muslim affiliation, was defined by the proportion of the population in the country of emigration (first generation) or a mother's country of emigration (second generation) who identifies as Muslim. This was determined from the Pew Research Centre (eTable 2).^18^ There were three exposure groups defined as (1) Muslim majority country (>50% identify as Muslim), (2) moderate proportion Muslim country (≥10% to ≤50% identify as Muslim) and (3) Muslim minority country (<10% identify as Muslim). Similar assignment of each country to a Muslim proportion has been used in other studies, as individual-level measures of religion are not measured or available across the population^19‐21^ and groupings were congruent with proportions identifying as Muslim from the United Nations Demography Yearbook.^22^

A secondary exposure was generation status with first-generation immigrants not born in Canada and arriving after their birth date, and second-generation immigrants born in an Ontario hospital with a mother with an IRCC immigration record from or after 1985. While paternal linkage has potential to provide an even deeper understanding of generational effects on immigration, paternal linkage and immigration records are not available in existing datasets. Other secondary exposures included (1) visa class: refugee, economic class, family class, or other^16^ and (2) age at immigration based on the arrival date in Canada.

### Study Outcomes

The primary outcome measures were (1) suicide deaths (between January 2003 and December 2018) and (2) emergency department visits for intentional self-harm (between January 2003 and March 2020). Suicide was determined from validated codes using the Ontario Registrar General – Deaths Vital Statistics file where the cause of death was from suicide (ICD-9 codes: E950-E959).^23^ Intentional self-harm was ascertained through the National Ambulatory Care Reporting System database with an International Classification of Diseases 10^th^ version with Canadian Modifications (ICD-10-CA) discharge code of X60-X84, Y10-Y19, or Y28 and included both self-harm with and without intent to die and undetermined intent (eTable 3).

### Covariates

Given the expected differences in outcomes by sex, all analyses were sex-stratified. Importantly, available datasets do not measure gender and therefore gender was not included as a covariate. Other covariates included neighborhood-level income quintile, neighbourhood-level material deprivation quintile, Canadian language ability at arrival in Canada, and country of birth.

### Statistical Analysis

We used frequencies and percentages to describe the baseline characteristics of immigrants from each of the three Muslim proportion country groups, stratified by sex. For each of the exposure groups, we calculated the rates of suicide (per 100,000 person-years) and intentional self-harm presentations (per 10,000 person-years) overall and within the sociodemographic characteristics. We used logistic regression to calculate the odds of death by suicide stratified by sex, adjusted for immigrant generation and the proportion of Muslims in the country of emigration. Negative binomial regression models were used to examine the relationship of the proportion of Muslims in the country of emigration and intentional self-harm, stratified by sex, controlling for refugee status, and age at immigration. We created funnel plots to examine rates of self-harm by individual country of origin for countries with at least 2,500 person-years of data.

Statistical analyses were conducted using SAS statistical software, version 9.4. Research Ethics Board approval was obtained from the Centre for Addiction and Mental Health Research Institute and the Sickkids Research Institute, both in Toronto, Canada. Cell sizes <6 were suppressed to meet institutional policy.

## Results

There were 1,070,248 first- and second-generation immigrant youth ages 12 to 24 years between 1 January 2003 and 31 May 2017 (**Table 1**). Half (50.1%) were female with 23.8% (129,919) females from Muslim majority countries and 24.2% (129,446) males from Muslim majority countries. Almost all (98–99%) lived in urban settings and immigrants from Muslim majority countries had the highest proportions living in the most deprived and lowest income neighbourhoods. For both males and females, immigrants from Muslim majority countries had the greatest proportion of refugees (29.1% [males], 26.4% [females]). First generation immigrants comprised 71.3% to 80.6% of the cohort and approximately one-quarter immigrated between the ages of 6 to 12 years. Immigrants from Muslim minority countries had the highest proportion with a Canadian language (English and/or French) ability at arrival.

**Table 1.** Baseline Demographic Characteristics of Immigrant Youth in Ontario, Canada by sex and by Proportion of the Population in the Country of Emigration That Identifies as Muslim.

	Male			Female		
	Muslim Population >50%	Muslim Population 10–50%	Muslim Population <10%	Muslim Population >50%	Muslim Population 10–50%	Muslim Population <10%
	N = 129,446	N = 90,715	N = 314,140	N = 129,919	N = 91,212	N = 314,816
Age at index, mean (SD)	15.0 (3.9)	15.2 (4.3)	15.2 (4.1)	15.6 (4.3)	15.9 (4.6)	15.6 (4.3)
Age at immigration, n (%)						
0–5	21,652 (16.7)	12,537 (13.8)	49,367 (15.7)	20,209 (15.6)	11,376 (12.5)	47,828 (15.2)
6–12	37, 389 (28.9)	20,872 (23.0)	80,669 (25.7)	33,975 (26.2)	18,387 (20.2)	77,010 (24.5)
13–17	22,712 (17.5)	13,345 (14.7)	46,079 (14.7)	19,785 (15.2)	11,657 (12.8)	42,424 (13.5)
18–24	21,266 (16.4)	17,904 (19.7)	42,605 (13.6)	30,710 (23.6)	25,777 (28.3)	57,174 (18.2)
Canada-born^a^	26,427 (20.4)	26,057 (28.7)	95,420 (30.4)	25,240 (19.4)	24,015 (26.3)	90,380 (28.7)
Immigration status, n (%)						
Economic	63,946 (49.4)	43,485 (47.9)	143,990 (45.8)	58,130 (44.7)	38,083 (41.8)	138,671 (44.0)
Family class	26,606 (20.6)	36,761 (40.5)	115,366 (36.7)	36,240 (27.9)	43,095 (47.2)	124,635 (39.6)
Refugees	37,618 (29.1)	9,858 (10.9)	49,796 (15.9)	34,345 (26.4)	9,455 (10.4)	46,388 (14.7)
Other immigrants	1,275 (1.0)	611 (0.7)	4,985 (1.6)	1,204 (0.9)	579 (0.6)	5,122 (1.6)
Generation, n (%)						
First	103,019 (79.6)	64,658 (71.3)	218,720 (69.6)	104,679 (80.6)	67,197 (73.7)	224,436 (71.3)
Second^a^	26,427 (20.4)	26,057 (28.7)	95,420 (30.4)	25,240 (19.4)	24,015 (26.3)	90,380 (28.7)
Canadian Language Ability, n (%)						
Yes	58510 (45.2)	43714 (48.2)	172520 (54.9)	59856 (46.1)	46263 (50.7)	175840 (55.9)
No	70936 (54.8)	47001 (51.8)	141620 (45.1)	70063 (53.9)	44949 (49.3)	138976 (44.1)
Neighbourhood income quintile, n (%)						
1 (lowest)	49,863 (38.5)	28,482 (31.4)	92,582 (29.5)	50,107 (38.6)	29,091 (31.9)	93,685 (29.8)
2	25,775 (19.9)	21,338 (23.5)	71,633 (22.8)	26,095 (20.1)	21,715 (23.8)	71,968 (22.9)
3	22,154 (17.1)	19,346 (21.3)	59,209 (18.8)	22,140 (17.0)	19,055 (20.9)	59,293 (18.8)
4	19,719 (15.2)	13,452 (14.8)	51,295 (16.3)	19,632 (15.1)	13,418 (14.7)	50,806 (16.1)
5 (highest)	11,740 (9.1)	7,986 (8.8)	38,918 (12.4)	11,756 (9.0)	7,806 (8.6)	38,573 (12.3)
Missing	195 (0.2)	111 (0.1)	503 (0.2)	189 (0.1)	127 (0.1)	491 (0.2)
Material deprivation quintile, n (%)						
1 (least deprived)	15,901 (12.3)	10,559 (11.6)	44,761 (14.2)	16,038 (12.3)	10,563 (11.6)	45,014 (14.3)
2	17,068 (13.2)	12,586 (13.9)	47,678 (15.2)	17,208 (13.2)	12,534 (13.7)	47,328 (15.0)
3	20,626 (15.9)	16,356 (18.0)	54,188 (17.2)	20,505 (15.8)	16,505 (18.1)	54,089 (17.2)
4	24,899 (19.2)	22,318 (24.6)	67,649 (21.5)	25,106 (19.3)	22,243 (24.4)	67,992 (21.6)
5 (most deprived)	49,910 (38.6)	28,179 (31.1)	97,192 (30.9)	50,073 (38.5)	28,627 (31.4)	97,803 (31.1)
Missing	1,042 (0.8)	717 (0.8)	2,672 (0.9)	989 (0.8)	740 (0.8)	2,590 (0.8)
Rurality, n (%)						
Urban	129,060 (99.7)	90,296 (99.5)	308,437 (98.2)	129,518 (99.7)	90,752 (99.5)	309,305 (98.2)
Rural	313 (0.2)	379 (0.4)	5,506 (1.8)	325 (0.3)	413 (0.5)	5,338 (1.7)
Missing	73 (0.1)	40 (0.0)	197 (0.1)	76 (0.1)	47 (0.1)	173 (0.1)

^a^ Country Muslim proportion determined based on maternal-child linkage and maternal country of birth.

### Suicide

Frequency and rates of suicide (per 100,000 person-years) stratified by sex and by proportion of the population in the country of emigration that identified as Muslim are presented in eTable 4. Across all exposure groups, males had higher suicide rates than females. Males from Muslim-majority countries had lower suicide rates (3.8 per 100,000 person years, 95% CI 2.7–5.4) compared to males from Muslim minority countries (5.9 per 100,000 person years, 95% CI 5.0–7.0) (adjusted odds ratio [OR]: 0.62, 95% CI 0.42–0.92), a finding unchanged when adjusting for first- and second-generation immigrant status. Immigrants from moderate proportion Muslim countries had suicide rates not different from those from Muslim minority countries. The number and rates of suicide among all female immigrants were low with rates not different by proportion Muslim in the country of emigration or by generation (eTable 4 and Table 2).

**Table 2.** Risk of Suicide in Immigrants by Sex and by Proportion Muslim in Country of Emigration.

	N With an Outcome	Rate per 100,000 Person-Years (95% CI)	Unadjusted OR (95% CI)	Adjusted OR (95% CI)^a^
Male				
Proportion Muslim				
>50%	33	3.8 (2.7–5.4)	0.64 (0.43–0.93)	0.62 (0.42–0.92)
10–50%	37	6.3 (4.6–8.7)	1.02 (0.71–1.47)	1.01 (0.70–1.46)
<10%	126	5.9 (5.0–7.0)	[Referent]	[Referent]
Generation				
First	148	5.9 (5.0–7.0)	–	[Referent]
Second	48	4.4 (3.3–5.9)	–	0.82 (0.59–1.13)
Female				
Proportion Muslim				
>50%	15	1.8 (1.1–3.0)	0.81 (0.45–1.45)	0.82 (0.46–1.47)
10–50%	9	1.6 (0.9–3.2)	0.69 (0.34–1.41)	0.69 (0.34–1.42)
<10%	45	2.2 (1.6–2.9)	[Referent]	[Referent]
Generation				
First	49	2.0 (1.5–2.7)	–	[Referent]
Second	20	2.0 (1.3–3.0)	–	1.14 (0.68–1.92)

CI = confidence interval; OR = odds ratio.

^a^ Adjusts for generation status.

### Intentional Self-Harm

The frequency and rates of hospital presentations for intentional self-harm (per 10,000 person-years), stratified by sex and by proportion of the population in the country of emigration that identified as Muslim are presented in eTable 5. In general, rates of intentional self-harm presentation among females were approximately triple those of males. Among males from Muslim majority countries, rates of intentional self-harm presentations were 12.2 per 10,000 person years (95% CI 11.5–13.1) (adjusted rate ratio [RR] 0.82, 95% CI 0.75, 0.90), and lowest in those from moderate proportion Muslim countries (9.6 per 10,000 person-years [95% CI 8.8–10.5]) (adjusted RR 0.67, 95% CI 0.60–0.75) and highest in males from Muslim minority (<10%) countries (14.1 per 10,000 person-years [95% CI 13.6–14.6]) (referent) (eTable 5). Refugees had higher rates of self-harm presentations compared with non-refugee immigrants (aRR 1.33, 95% CI 1.22–146) and among males there were no differences in self-harm presentation rates by age at immigration (Table 3).

**Table 3.** Risk of Self-harm Presentation in Immigrants by Sex and by Proportion Muslim in Country of Emigration.

	N With an Outcome	Rate per 10,000 Person-Years (95% CI)	Unadjusted RR (95% CI)	Adjusted RR (95% CI)^a^
Male				
Proportion Muslim				
>50%	926	12.2 (11.5–13.1)	0.85 (0.79–0.94)	0.82 (0.75–0.90)
10–50%	489	9.6 (8.8–10.5)	0.66 (0.60–0.74)	0.67 (0.60–0.75)
<10%	2664	14.1 (13.6–14.6)	[Referent]	[Referent]
Refugee status				
Refugee	939	16.2 (15.2–17.3)	–	1.33 (1.22–1.46)
Non-refugee	3140	12.2 (11.8–12.6)	[Referent]	[Referent]
Age at immigration				
0–5	790	13.1 (12.2–14.1)	–	1.03 (0.92–1.15)
6–12	1250	12.7 (12.0–14.1)	–	1.05 (0.95–1.16)
13–17	631	13.2 (12.2–14.3)	–	1.08 (0.96–1.21)
18–24	287	13.2 (11.8–14.9)	–	1.04 (0.90–1.20)
Canadian born	1121	12.8 (12.1–13.6)	[Referent]	[Referent]
Female				
Proportion Muslim				
>50%	2158	30.1 (28.9–31.4)	0.92 (0.86–0.98)	0.93 (0.87–1.00)
10–50%	1161	24.2 (22.8–25.6)	0.73 (0.67–0.79)	0.77 (0.71–0.83)
<10%	6009	32.8 (32.0–33.6)	[Referent]	[Referent]
Refugee status				
Refugee	2096	38.9 (37.1–40.4)	–	1.37 (1.27–1.46)
Non-refugee	7232	29.1 (28.4–29.7)	[Referent]	[Referent]
Age at immigration				
0–5	2045	35.4 (33.9–37.0)	–	0.92 (0.85–0.99)
6–12	2433	26.5 (25.5–27.6)	–	0.69 (0.64–0.74)
13–17	1289	30.0 (28.4–31.7)	–	0.74 (0.72–0.86)
18–24	583	21.0 (19.3–22.8)	–	0.54 (0.49–0.60)
Canadian born	2978	35.9 (34.6–37.2)	[Referent]	[Referent]

^a^ Model adjusted for refugee status and age at immigration.

Among female immigrants, rates of intentional self-harm presentations were not different between Muslim majority and Muslim minority (<10%) countries. However, age at immigration was inversely related to risk of self-harm with an older age (vs. younger age) at immigration conferring a lower relative risk of self-harm (eTable 5).

**Figure 1.**
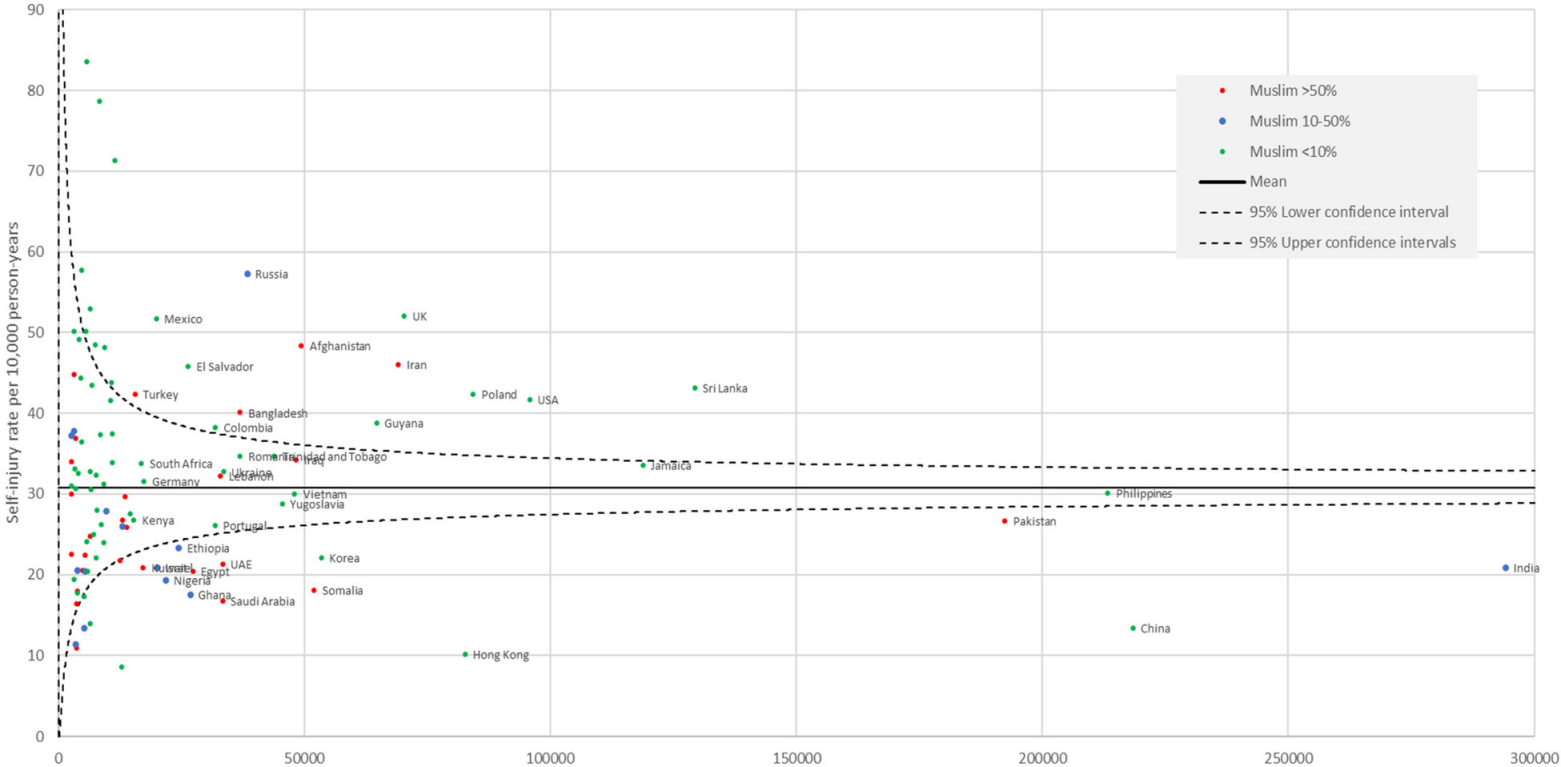
Rate of self-harm presentations among females by country. Only countries with  > 2,500 person-years of follow up included and only those with >15,000 person-years labeled. The reference is the sex-specific mean immigrant rate and 95% confidence intervals.

**Figure 2.**
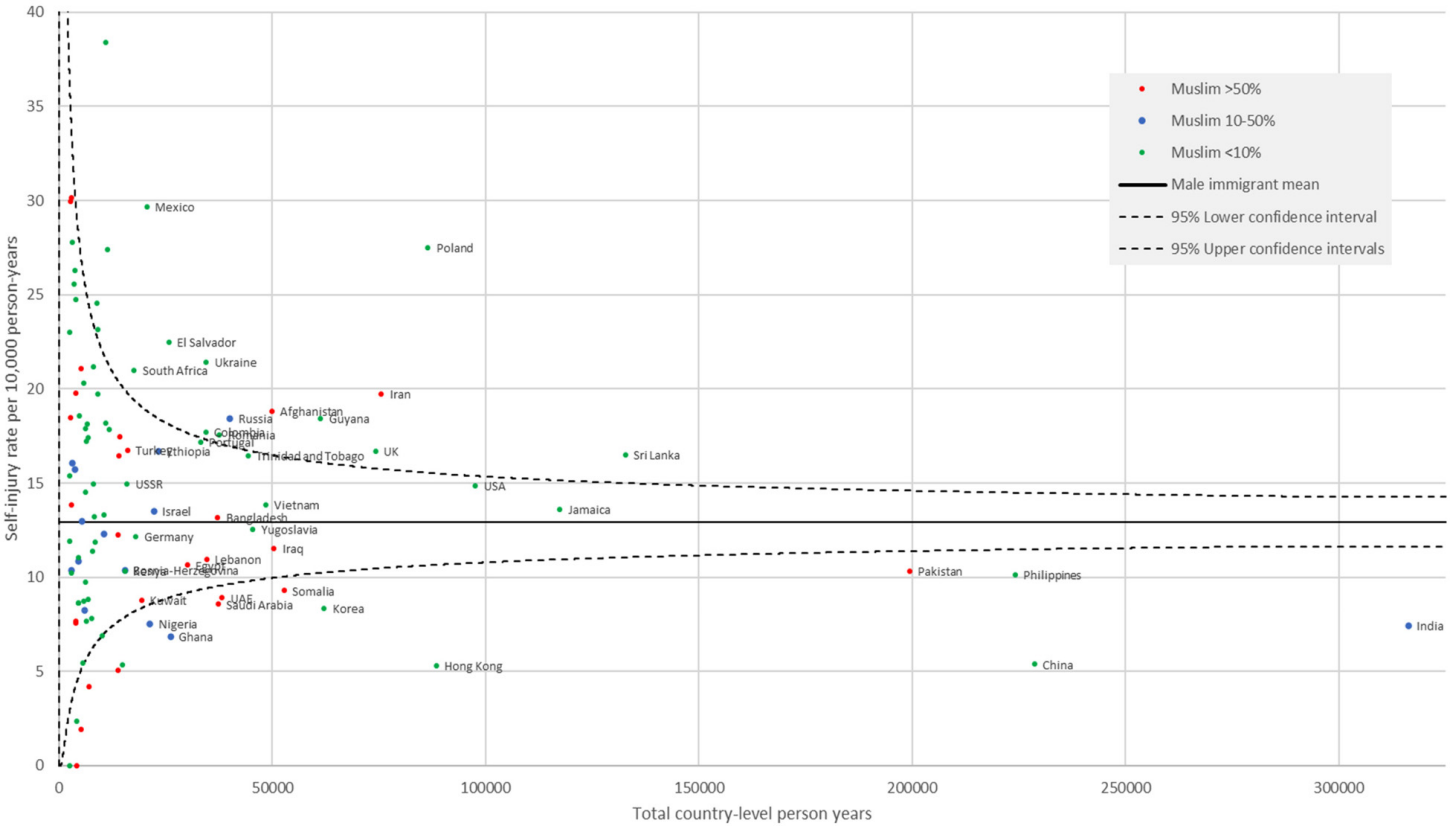
Rate of self-harm presentations among males by country. Only countries with  > 2,500 person-years of follow up included and only those with >15,000 person-years labeled. The reference is the sex-specific mean immigrant rate and 95% confidence intervals.

Country-specific intentional self-harm presentation rates by sex and by person-years of data are shown in Figure 1 (females) and Figure 2 (males) with a reference line showing the overall sex-specific immigrant self-harm rate and 95% confidence intervals. Among high-volume countries, both males and females from Afghanistan, Iran, Sri Lanka, United Kingdom, Poland, Russia, Guyana, Mexico and El Salvador had high rates of intentional self-harm and those from Pakistan, Somalia, Saudi Arabia, the United Arab Emirates, India, China, Hong Kong, and Korea had low rates of intentional self-harm compared to the mean sex-specific immigrant rates. Of the Muslim majority countries, females but not males from Bangladesh and Turkey had higher rates than the mean female immigrant rate.

## Discussion

In this population-based cohort study of immigrant youth in Ontario, Canada, we showed that the suicide rate in males from Muslim majority countries was approximately 40% lower than that of immigrants from countries with a small minority (<10%) of Muslims. In contrast, suicide rates among female immigrants from Muslim majority countries were comparable to those from countries with a low proportion of Muslims. Similarly, immigrant males from Muslim majority countries had lower visit rates for intentional self-harm presentations compared with those from Muslim minority countries, a difference not observed among females. Other notable findings include no difference in the odds of dying by suicide in first- and second-generation immigrants and that risk of self-harm presentations decreased with increasing age at immigration among female but not male immigrants.

Religious beliefs and pre-migration experiences (e.g., war, genocide) and environment related to attitudes towards and laws about suicide, the value of religion, and religious practices may contribute to our findings.^16^ Those who endorse religious beliefs that disapprove of suicide have fewer suicide attempts.^24,25^ Religion may buffer against distress and encourage self-regulation, social support and positive coping. Islamic tradition consistently denounces suicide, with many interpretations of the Qu’ran explicitly forbidding a person to end their life. Similar pronouncements in other religions, including Catholicism and Judaism, exist and have been noted to be protective.^7,16^ In many Muslim majority countries and regions (e.g., Saudi Arabia, Pakistan, Kuwait), suicide and suicide attempts are considered criminal offences^26,27^ and in this study, immigrants from these countries had particularly low rates of self-harm.

Survey studies indicate that Muslim youth struggle with suicidal ideation across the globe.^28,29^ A previous study of Pakistani adolescents reported higher rates of suicidal ideation and behaviours compared to other ethnicities studied and a study from the Netherlands reported disproportionately high rates of self-reported suicidal behaviour or ideation among young women in the Netherlands who were of Turkish, Moroccan, and South-Asian-Surianamese descent.^28,29^ In the United States, those identifying as Muslim (all ages) had self-reported lifetime suicide attempts twice as high as other religious groups, albeit with much lower attempts in those born outside of the United States.^9^ In contrast, a United Kingdom study found no differences in the rates of suicidal thoughts, plans or behaviours between young Muslims and Hindus in the Greater London Area.^30^ Yet, we report at a population level, that immigrants from Muslim majority countries had rates of intentional self-harm presentations or suicide not different (females) or lower (males) than those from Muslim minority countries. The variable findings between our study and others suggest jurisdictional context and culture, in addition to immigration may be important in understanding the risk or protective effect of Muslim affiliation and suicide risk.

Importantly, we show large differences in these groups by sex, with males from Muslim majority countries seemingly protected for self-harm presentations, an advantage not conferred to females. Our findings point to the importance of stratified analyses and potentially different preventive approaches when considering the relationship between religious affiliation and suicide risk for males versus females. In Ontario immigrant youth, the extent to which they participate in, or identify with their religious community, experience gender-based differences in roles and ways of relating to religion and religious communities, or experience Islamophobia may vary by sex^15,31^ which, in turn, may reflect the differential sex-based findings in our study. Prior studies^32^ have shown that among males, there is an inverse relationship between suicide rates and religious belief and attendance. However, this relationship has not been established in females. Other studies^33^ report higher rates of suicide in men (compared to women) who identify as Christian, Hindu or Jewish but similar rates of suicide in men and women who identify as Muslim.

We report sex differences in time since immigration and self-harm presentation risk. Societal secularization, settlement and integration may change the role of religion and pre-migration attitudes and beliefs towards suicide, especially as immigrants stay in Canada. For females, this may be more pronounced than among males and may explain the increasing rates of self-harm in females not observed in males with younger ages at immigration. This is important in the context of immigration, where providing access to a supportive community, especially for female immigrants, refugees, and those from Muslim majority countries, may be helpful in reducing suicide risk. Indeed, the current findings echo our previous work demonstrating the increased suicide and self-harm risk of refugees compared to other immigrant groups and highlights an opportunity for ensuring access to evidence-based suicide prevention strategies, including dialectical behaviour therapy and family-centred therapy, for these populations.^2,4,34^ The generational and time since immigration findings of our study point to the importance of understanding cultural versus immigration specific nuances to suicide risk, as the protective effects of Muslim affiliation we observed have not consistently been observed across other jurisdictions that have been studied.^9,28,29^

### Strengths and Limitations

Strengths of this study include the use of validated codes for suicide^23^ and a large sample size with almost complete provincial coverage of the immigrant population in Ontario. We included detailed immigration information with systematic and uniform data collection that does not rely on self-reporting. There are some important limitations. We measure self-harm presentations based on a visit to an acute care centre. This does not include those with suicidal ideation or milder physical injuries not requiring medical care. Immigrants to Canada utilize mental health services at lower rates than Canadian-born^4^ and therefore acute care presentations for self-harm may underrepresent true community rates. Using health administrative data, we cannot identify the motivation for self-harm (i.e., whether and to what degree there may have been suicidal intent). We do not measure community and social supports, including those from allied health (social workers, psychologists), settlement or religious supports nor do we have measures of religious affiliation or religiosity. While findings apply to immigrants from Muslim majority countries, our measure of Muslim affiliation is ecological in that we are assigning Muslim affiliation to individuals on the basis of Muslim population proportion from the country of origin. As such, we are likely underestimating the effect of Muslim affiliation. There is also important heterogeneity in practices, beliefs and experiences within and outside countries of origin and variation the role religion plays as a cultural and/or a spiritual construct for individuals. Qualitative work, including those with lived experience, will likely help to give dimension to the current findings and fill these gaps. Refugees to Canada receive provincial health insurance on arrival and non-refugee immigrants receive this within three months. Our study does not include temporary or undocumented immigrants and refugees seeking asylum without permanent residency or second-generation immigrants based on their father's immigration status as they are not currently linkable with existing datasets.

## Conclusions

Immigrants to Ontario, Canada from Muslim majority countries have low rates of suicide and self-harm presentations to acute care centres among males but not females, compared to those from Muslim minority countries. Suicide prevention strategies may consider the potential that Muslim affiliation may be protective in males, but further research investigating the sex-based differences observed among female immigrants is warranted. This mixed methods study's qualitative research arm will aim to explore and address these issues.

## Supplemental Material

sj-docx-1-cpa-10.1177_07067437231166840 - Supplemental material for Suicide and Self-Harm Among Immigrant Youth to Ontario, Canada From Muslim Majority Countries: A Population-Based Study Click here for additional data file. Supplemental material, sj-docx-1-cpa-10.1177_07067437231166840 for Suicide and Self-Harm Among Immigrant Youth to Ontario, Canada From Muslim Majority Countries: A Population-Based Study by Natasha Saunders, Rachel Strauss, Sarah Swayze, Alex Kopp, Paul Kurdyak, Zainab Furqan, Arfeen Malick, Muhammad Ishrat Husain, Mark Sinyor and Juveria Zaheer in The Canadian Journal of Psychiatry

## Abbreviations

CI: confidence interval
CIHI DAD: Canadian Institutes of Health Information Discharge Abstract Database Immigration
IRCC: Immigration, Refugees and Citizenship Canada
ICD-10-CA: International Classification of Diseases 10^th^ Revision
NACRS: National Ambulatory Care Reporting System
OHIP: Ontario Health Insurance Plan
RPDB: Registered Persons Database.
