# Supplementary material for: Suicide and Self-Harm Among Immigrant Youth to Ontario, Canada From Muslim Majority Countries: A Population-Based Study
Source: Can J Psychiatry. 2023 Apr 10;68(10):755–65. doi: 10.1177/07067437231166840 (PMC10517651; doi:10.1177/07067437231166840)

**Supplemental Files**

**eTable 1.** Data sources and study variables

| **Data source** | **Years** | **Variables** |
| --- | --- | --- |
| Canadian Institute for Health Information Discharge Abstract Database (CIHI-DAD) | 2003-2020 | Hospital admissions |
| National Ambulatory Care Reporting System (NACRS) | 2003-2020 | ED visits for self-harm |
| Ontario Registrar General Vital Statistics—Deaths (ORGD) | 2003-2018 | Suicides |
| ICES Mother-Baby Linked Database (MOMBABY) | All years | Record of all mothers linked to their Ontario-born children |
| Registered Person Database (RPDB) | All years | Patient sex, residential postal code, date of birth |
| Immigration, Refugees and Citizenship Canada's Permanent Resident Database | 2003-2017 | Immigration status; country of birth; age at immigration; immigration generation; Canadian language ability |
| Ontario Marginalization Index | Latest (2016) | Material deprivation quintile |
| 2016 Canadian Census |  | Rurality |
| Canada’s Postal Code Con- version File (PCCF) |  |  |

**eTable 2.** List of countries by proportion of population identifying as Muslim

| **Muslim-majority (>50%)** | | **Muslim population between 10 and <50%** | **Muslim-minority (<10% Muslim)** | | | |
| --- | --- | --- | --- | --- | --- | --- |
| Afghanistan | Mauritania | Benin | Andorra | Congo | Latvia | New Zealand |
| Albania | Mayotte | Bosnia-Herzegovina | Angola | Croatia | Liechtenstein | Northern Mariana Islands |
| Algeria | Morocco | Bulgaria | Anguilla | Cuba | Lithuania | Slovakia |
| Azerbaijan | Niger | Cameroon | Antigua and Barbuda | Denmark | Panama | Slovenia |
| Bahrain | Oman | Cyprus | Argentina | Dominica | Philippines | South Africa |
| Bangladesh | Pakistan | Eritrea | Aruba | Equatorial Guinea | Poland | South Korea |
| Brunei | Palestine territories | Ethiopia | Australia | Estonia | Portugal | Spain |
| Burkina Faso | Qatar | Georgia | Austria | Fiji | Republic of Congo | Sri Lanka |
| Chad | Saudi Arabia | Ghana | Bahamas | Finland | Reunion | St. Kitts and Nevis |
| Comoros | Senegal | Guinea Bissau | Barbados | France | Romania | St. Lucia |
| Djibouti | Sierra Leone | India | Belarus | French Guiana | Rwanda | St. Pierre and Miquelon |
| Egypt | Somalia | Israel | Belgium | Gabon | Serbia | St. Vincent and the Grenadines |
| Gambia | Sudan | Ivory Coast | Belize | Germany | Seychelles | Swaziland |
| Guinea | Syria | Liberia | Bermuda | Gibraltar | Luxembourg | Sweden |
| Indonesia | Tunisia | Malawi | Bhutan | Greece | Madagascar | Switzerland |
| Iran | Turkey | Mauritius | Botswana | Grenada | Malta | Taiwan |
| Iraq | Turkmenistan | Montenegro | Brazil | Guadeloupe | Martinique | Thailand |
| Jordan | United Arab Emirates | Mozambique | British Virgin Islands | Guyana | Mexico | Timor-Leste |
| Kazakhstan | Uzbekistan | Nigeria | Burma (Myanmar) | Honduras | Moldova | Trinidad and Tobago |
| Kosovo | Western Sahara | Republic of Macedonia | Burundi | Hong Kong | Monaco | Tuvalu |
| Kuwait | Yemen | Russia | Cambodia | Hungary | Mongolia | U.S. Virgin Islands |
| Kyrgyzstan |  | Singapore | Canada | Iceland | Montserrat | Ukraine |
| Lebanon |  | Suriname | Cape Verde | Ireland | Namibia | United Kingdom |
| Libya |  | Tanzania | Cayman Islands | Isle of Man | Nepal | United States |
| Malaysia |  | Togo | Central African Republic | Italy | Netherlands | Venezuela |
| Maldives |  | Uganda | Channel Islands | Japan | Netherlands Antilles | Vietnam |
| Mali |  |  | China | Kenya | New Caledonia | Zambia |

**eTable 3.** List of diagnostic codes

| **Death by suicide** | | |
| --- | --- | --- |
| **Data Source** | **ICD-9 Diagnostic Code** | **Description** |
| **Ontario Registrar General Vital Statistics—Deaths (ORGD)** | E950 | Suicide and self-inflicted poisoning by solid or liquid substances |
|  | E951 | Suicide and self-inflicted poisoning by gases in domestic use |
|  | E952 | Suicide and self-inflicted poisoning by other gases and vapors |
|  | E953 | Suicide and self-inflicted injury by hanging strangulation and suffocation |
|  | E954 | Suicide and self-inflicted injury by submersion [drowning] |
|  | E955 | Suicide and self-inflicted injury by firearms air guns and explosives |
|  | E956 | Suicide and self-inflicted injury by cutting and piercing instrument |
|  | E957 | Suicide and self-inflicted injuries by jumping from high place |
|  | E958 | Suicide and self-inflicted injury by other and unspecified means |
|  | E959 | Late effects of self-inflicted injury |
| **Intentional self-harm** | | |
| **Data Source** | **ICD-10 Disagnostic Code** | **Description** |
| **National Ambulatory Care Reporting System database (NACRS)** | X60 | Intentional self-poisoning by and exposure to nonopioid analgesics, antipyretics, and antirheumatics |
|  | X61 | Intentional self-poisoning by and exposure to antiepileptic, sedative-hypnotic, antiparkinsonism, and psychotropic drugs, not elsewhere classified |
|  | X62 | Intentional self-poisoning by and exposure to narcotics and psychodysleptics [hallucinogens], not elsewhere classified |
|  | X63 | Intentional self-poisoning by and exposure to other drugs acting on the autonomic nervous system |
|  | X64 | Intentional self-poisoning by and exposure to other and unspecified drugs, medicaments, and biological substances |
|  | X65 | Intentional self-poisoning by and exposure to alcohol |
|  | X66 | Intentional self-poisoning by and exposure to organic solvents and halogenated hydrocarbons and their vapours |
|  | X67 | Intentional self-poisoning by and exposure to other gases and vapours |
|  | X68 | Intentional self-poisoning by and exposure to pesticides |
|  | X69 | Intentional self-poisoning by and exposure to other and unspecified chemicals and noxious substances |
|  | X70 | Intentional self-harm by hanging, strangulation, and suffocation |
|  | X71 | Intentional self-harm by drowning and submersion |
|  | X72 | Intentional self-harm by handgun discharge |
|  | X73 | Intentional self-harm by rifle, shotgun, and larger firearm discharge |
|  | X74 | Intentional self-harm by other and unspecified firearm discharge |
|  | X75 | Intentional self-harm by explosive material |
|  | X76 | Intentional self-harm by smoke, fire, and flames |
|  | X77 | Intentional self-harm by steam or hot vapors |
|  | X78 | Intentional self-harm by sharp object |
|  | X80 | Intentional self-harm by jumping from a high place |
|  | X81 | Intentional self-harm by jumping or lying before moving object |
|  | X82 | Intentional self-harm by crashing of motor vehicle |
|  | X83 | Intentional self-harm by other specified means |
|  | X84 | Intentional self-harm by unspecified means |
|  | Y10 | Poisoning by and exposure to nonopioid analgesics, antipyretics and antirheumatics, undetermined intent |
|  | Y11 | Poisoning by and exposure to antiepileptic, sedative-hypnotic, antiparkinsonism and psychotropic drugs, not elsewhere classified, undetermined intent |
|  | Y12 | Poisoning by and exposure to narcotics and psychodysleptics [hallucinogens], not elsewhere classified, undetermined intent |
|  | Y13 | Poisoning by and exposure to other drugs acting on the autonomic nervous system, undetermined intent |
|  | Y14 | Poisoning by and exposure to other and unspecified drugs, medicaments and biological substances, undetermined intent |
|  | Y15 | Poisoning by and exposure to alcohol, undetermined intent |
|  | Y16 | Poisoning by and exposure to organic solvents and halogenated hydrocarbons and their vapours, undetermined intent |
|  | Y17 | Poisoning by and exposure to other gases and vapours, undetermined intent |
|  | Y18 | Poisoning by and exposure to pesticides, undetermined intent |
|  | Y19 | Poisoning by and exposure to other and unspecified chemicals and noxious substances, undetermined intent |
|  | Y28 | Contact with sharp object, undetermined intent |

**eTable 4:** Number and rates of suicide per 100,000 person-years by sociodemographic characteristics of immigrant youth in Ontario, Canada by sex and by proportion of the population in the country of emigration that identifies as Muslim.

|  | **Male** | | | | | | **Female** | | | | | |
| --- | --- | --- | --- | --- | --- | --- | --- | --- | --- | --- | --- | --- |
|  | **Muslim**  **Population >50%** N=129,446 | | **Muslim**  **Population 10-50%** N=90,715 | | **Muslim**  **Population <10%** N=314,140 | | **Muslim**  **Population >50%** N=129,919 | | **Muslim**  **Population 10-50%** N=91,212 | | **Muslim**  **Population <10%** N=314,816 | |
| Socio-demographic variable | N | Rate/100K (95%CI) | N | Rate/100K (95%CI) | N | Rate/100K (95%CI) | N | Rate/100K (95%CI) | N | Rate/100K (95%CI) | N | Rate/100K (95%CI) |
| **Age at immigration, n (%)**  0-5  6-12  13-17  18-24  Canadian-born | 9  11  6  -  - | 5.2 (2.7-9.9)  3.6 (2.0-6.5)  4.1 (1.9-9.2)  -  - | 5-10  10  7  -  11 | 7.4 (3.5-15.5)  5.6 (3.0-10.5)  8.0 (3.8-16.8)  -  6.1 (3.4-11.0) | 20  41  21  12  32 | 4.9 (3.2-7.6)  6.9 (5.1-9.4)  7.5 (4.9-11.4)  9.8 (5.6-17.3)  4.4 (3.1-6.2) | -  -  -  -  - | 1.2 (0.3-4.9)  1.4 (0.5-3.9)  3.2 (1.2-8.4)  -  1.2 (0.3-4.8) | -  -  -  -  - | 1.2 (0.2-8.2)  1.9 (0.6-6.0)  2.6 (0.7-10.5)  -  1.2 (0.3-4.8) | 11  8  8  -  13-19 | 2.8 (1.5-5.0)  1.4 (0.7-2.8)  3.1 (1.5-6.2)  -  2.3 (1.4-3.8) |
| **Immigration status, n (%)** Economic immigrants  Family class immigrants  Refugees  Other immigrants | 17  -  11  - | 3.7 (2.3-6.0)  -  4.6 (2.5-8.3)  - | 16  14  7  0 | 5.4 (3.3-8.7)  6.5 (3.8-11.0)  10.5 (5.0-22.1)  0.0 (0.0-0.0) | 54  46  19-24  - | 5.4 (4.1-7.0)  6.1 (4.6-8.2)  6.4 (4.2-9.7)  - | 6  - 7  - | 1.5 (0.7-3.2)  -  3.1 (1.5-6.6)  - | -  -  -  - | 1.9 (0.8-4.6)  1.3 (0.4-4.2)  1.6 (0.2-11.3)  - | 18-23  17  6  - | 2.2 (1.4-3.4)  2.3 (1.4-3.6)  1.9 (0.8-4.1)  - |
| **Immigrant generation, n (%)**  First  Second | 28-33  - | 4.1 (2.8-5.9)  - | 26  11 | 6.4 (4.4-9.4)  6.1 (3.4-11.0) | 94  32 | 6.7 (5.5-8.2)  4.4 (3.1-6.2) | 10-15  - | 2.0 (1.2-3.5)  1.2 (0.3-4.8) | 5-9  - | 1.8 (0.9-3.8)  - | 29  16 | 2.1 (1.5-3.0)  2.3 (1.4-3.8) |
| **Canadian Language Ability, n (%)** Yes  No | 15  18 | 4.2 (2.5-7.0)  3.6 (2.3-5.7) | 10  27 | 4.0 (2.2-7.5)  8.2 (5.6-12.0) | 70  56 | 6.2 (4.9-7.9)  5.6 (4.3-7.2) | 6  9 | 1.8 (0.8-3.9)  1.9 (1.0-3.6) | -  - | -  - | 19  26 | 1.7 (1.1-2.7)  2.7 (1.8-4.0) |
| **Neighbourhood income quintile, n (%)**  1 (lowest)  2  3  4  5 (highest) | 12  8  -  -  - | 3.6 (2.0-6.3)  4.7 (2.4-9.4)  -  -  - | 10  15  9  -  - | 5.4 (2.9-10.0)  11.1 (6.7-18.4)  7.3 (3.8-14.0)  -  - | 41  22  19-24  22  - | 6.6 (4.9-9.0)  4.5 (3.0-6.9)  5.2 (3.4-8.0)  6.2 (4.1-9.4)  - | 7  -  -  -  - | 2.2 (1.0-4.6)  -  -  -  - | -  - 0  -  - | -  - 0  -  - | 7  14  15  -  - | 1.2 (0.5-2.4)  3.0 (1.8-5.1)  3.8 (2.3-6.4)  -  - |
| **Material deprivation quintile, n (%)**  1 (least deprived)  2  3  4  5 (most deprived) | -  -  11  -  12 | -  -  8.1 (4.5-14.6)  -  3.6 (2.0-6.3) | -  -  11  12  9 | -  -  10.5 (5.8-19.0)  8.5 (4.8-15.0)  5.0 (2.6-9.6) | 21  15  13  35  42 | 6.6 (4.3-10.1)  4.6 (2.8-7.6)  3.5 (2.0-6.0)  7.7 (5.5-10.8)  6.5 (4.8-8.8) | -  -  -  -  6-10 | -  -  -  -  2.5 (1.2-5.0) | 0  0  -  -  - | 0.0 (0.0-0.0)  0.0 (0.0-0.0)  -  -  - | - 9  15  -  12 | -  2.9 (1.5-5.5)  4.2 (2.5-7.0)  -  1.9 (1.1-3.4) |
|  |  |  |  |  |  |  |  |  |  |  |  |  |
| **Rurality, n (%)** Urban  Rural | 33  0 | 3.8 (2.7-5.4)  0.0 (0.0-0.0) | 33-37  - | 6.0 (4.3-8.4)  - | 120-126  - | 5.9 (4.9-7.0)  - | 15  0 | 1.8 (1.1-3.1)  0.0 (0.0-00) | 9  0 | 1.6 (0.9-3.2)  0.0 (0.0-0.0) | 45  0 | 2.2 (1.7-3.0)  0.0 (0.0-0.) |

**eTable 5:** Number and rates of intentional self-harm presentations per 10,000 person-years by sociodemographic characteristics of immigrant youth in Ontario, Canada by sex and by proportion of the population in the country of emigration that identifies as Muslim.

|  | **Male** | | | | | | **Female** | | | | | |
| --- | --- | --- | --- | --- | --- | --- | --- | --- | --- | --- | --- | --- |
|  | **Muslim**  **Population >50%** N=129,446 | | **Muslim**  **Population 10-50%** N=90,715 | | **Muslim**  **Population <10%** N=314,140 | | **Muslim**  **Population >50%** N=129,919 | | **Muslim**  **Population 10-50%** N=91,212 | | **Muslim**  **Population <10%** N=314,816 | |
|  | N | Rate/10K (95%CI) | N | Rate/10K (95%CI) | N | Rate/10K (95%CI) | N | Rate/10K (95%CI) | N | Rate/10K (95%CI) | N | Rate/10K (95%CI) |
| **Age at immigration**  0-5  6-12  13-17  18-24  Canadian-born | 173  304  191 92  166 | 11.5 (9.9-13.3)  11.0 (9.8.-12.3)  14.3 (12.4-16.5)  16.0 (13.1-19.7)  12.1 (10.4-14.0) | 94  141  89  35  130 | 11.9 (9.7-14.5) 8.8 (7.4-10.3)  10.7 (8.7-13.2)  7.9 (5.7-11.1)  9.1 (7.7-10.9) | 523  805  351  160  825 | 14.1 (12.9-15.3)  14.7 (13.7-15.7)  13.4 (12.1-14.9)  13.9 (11.9-16.2)  13.9 (13.0-148.4) | 417  695  403  203  440 | 29.5 (26.8-32.5)  27.7 (25.7-29.8)  34.5 (31.3-38.1)  26.3 (22.9-30.1)  33.6 (30.6-36.9) | 256  303  168  111  323 | 35.1 (31.1-39.7)  21.3 (19.1-23.9)  23.2 (20.0-27.0)  18.3 (15.1-22.0)  24.4 (21.9-27.2) | 1372  1435  718  269  2215 | 37.8 (35.9-39.9)  27.4 (26.0-28.9)  29.9 (27.8-32.2)  19.2 (17.1-21.7)  39.2 (37.6-40.8) |
| **Immigration status**  Economic immigrants  Family class immigrants  Refugees  Other immigrants | 412  172  335  7 | 10.1 (9.2-11.1)  13.3 (11.4-15.4)  15.8 (14.2-17.6)  10.0 (4.8-21.1) | 208  205  68  8 | 7.9 (6.9-9.0)  11.2 (9.8-12.9)  11.6 (9.2-14.8)  21.2 (10.6-42.5) | 972  1111  536  45 | 10.8 (10.2-11.5)  16.9 (15.9-17.9)  17.4 (16.0-18.9)  15.6 (11.7-20.9) | 850  445  2176  821 | 23.3 (21.8-24.9)  29.9 (27.3-32.9)  41.9 (39.1-44.8)  59.8 (44.2-80.9) | 429  576  150  6 | 18.7 (17.0-20.5)  30.0 (27.6-32.5)  27.0 (23.0-31.7)  18.1 (8.2-40.4) | 2176  2588  1125  120 | 25.6 (24.6-26.7)  39.0 (37.5-40.5)  38.8 (36.6-41.1)  41.2 (34.5-49.3) |
| **Immigrant generation**  First  Second | 760  166 | 12.3 (11.4-13.2)  12.1 (10.4-14.0) | 359  130 | 9.8 (8.8-10.8)  9.1 (7.7-10.9) | 1839  825 | 14.2 (13.4-14.8)  13.9 (13.05-14.8) | 1718  440 | 29.3 (28.0-30.7)  33.6 (30.6-36.9) | 838  323 | 24.1 (22.5-25.8)  24.4 (21.9-27.2) | 3794  2215 | 30.0 (29.0-30.9)  39.2 (37.6-40.8) |
| **Canadian Language Ability**  Yes  No | 366  560 | 11.7 (10.5-12.9)  12.7 (11.6-13.7) | 201  288 | 9.0 (7.8-10.3)  10.1 (9.0-11.2) | 1458  1206 | 14.8 (14.1-15.6)  13.3 (12.6-14.1) | 875  1283 | 29.1 (27.2-31.1)  30.8 (29.2-32.5) | 461  700 | 21.2 (19.3-23.2)  26.6 (24.7-28.7) | 3584  2425 | 37.1 (35.9-38.3)  28.0 (26.9-29.1) |
| **Neighbourhood income quintile**  1 (lowest)  2  3  4  5 (highest) | 358  210  151  132  74 | 12.0 (10.8-13.3)  14.1 (12.3-16.1) 11.6 (9.9-13.6)  11.8 (10.0-14.0)  11.3 (9.0-14.2) | 152 90  106  58  81 | 9.3 (7.9-10.9)  7.6 (6.2-9.4)  10.0 (8.3-12.1)  7.7 (6.0-10.0)  17.8 (14.3-22.1) | 772  640  479  391  375 | 13.9 (13.0-15.0)  14.9 (13.8-16.1)  13.4 (12.3-14.7)  12.5 (11.4-13.9)  15.8 (14.3-17.5) | 825  440  389  280  222 | 29.0 (27.1-31.1)  30.9 (28.1-33.9)  32.1 (29.0-35.4)  26.3 (23.4-29.5)  35.9 (31.5-40.9) | 323  278  221  181  157 | 20.7 (18.6-23.1)  24.8 (22.1-27.9)  22.6 (19.8-25.7)  25.1 (21.7-29.0)  37.6 (32.1-43.9) | 1664  1356  1060  1042  880 | 30.6 (29.2-32.1)  32.7 (31.0-34.5)  30.7 (28.9-32.6)  34.7 (32.7-36.9)  38.9 (36.4-41.6) |
| **Material deprivation quintile**  1 (least deprived)  2  3  4  5 (most deprived) | 93  98  149  203  372 | 9.8 (8.0-12.0)  10.0 (8.2-12.2)  12.5 (10.7-14.7)  14.4 (12.5-16.5)  12.5 (11.3-13.9) | 83  71  105 91  132 | 13.1 (10.5-16.2)  9.8 (7.8-12.4)  11.6 (9.6-14.1)  7.5 (6.1-9.2)  8.4 (7.1-9.9) | 404  388  449  566  825 | 14.2 (12.9-15.6)  13.4 (12.2-14.8)  13.7 (12.5-15.0)  14.2 (13.1-15.4)  14.4 (13.4-15.4) | 253  298  334  443  819 | 28.1 (24.9-31.8)  32.3 (28.8-36.2)  30.0 (26.9-33.4)  33.0 (30.1-36.3)  28.9 (27.0-31.0) | 208  168  185  273  318 | 35.0 (30.5-40.1)  25.1 (21.5-29.2)  21.4 (18.6-24.8)  24.2 (21.5-27.2)  21.1 (18.9-23.6) | 938  923  970  1271  1831 | 34.0 (31.9-36.2)  33.3 (31.2-35.5)  30.6 (28.7-32.6)  32.9 (31.1-34.7)  32.8 (31.3-34.3) |
| **Rurality, n (%)** Urban  Rural | 925  - | 12.2 (11.5-13.1)  - | 481  6 | 9.5 (8.7-10.4)  30.2 (13.6-67.2) | 2600  60 | 14.0 (13.5-14.6)  16.9 (13.1-21.8) | 2145  12 | 30.0 (28.7-31.3)  82.7 (47.0-145.7) | 1148  12 | 24.0 (22.7-25.4)  58.5 (33.2-103.1) | 5854  149 | 32.6 (31.7-33.4)  44.6 (38.0-52.3) |

**eFigure 1**. Flow chart of study inclusion and exclusion criteria.


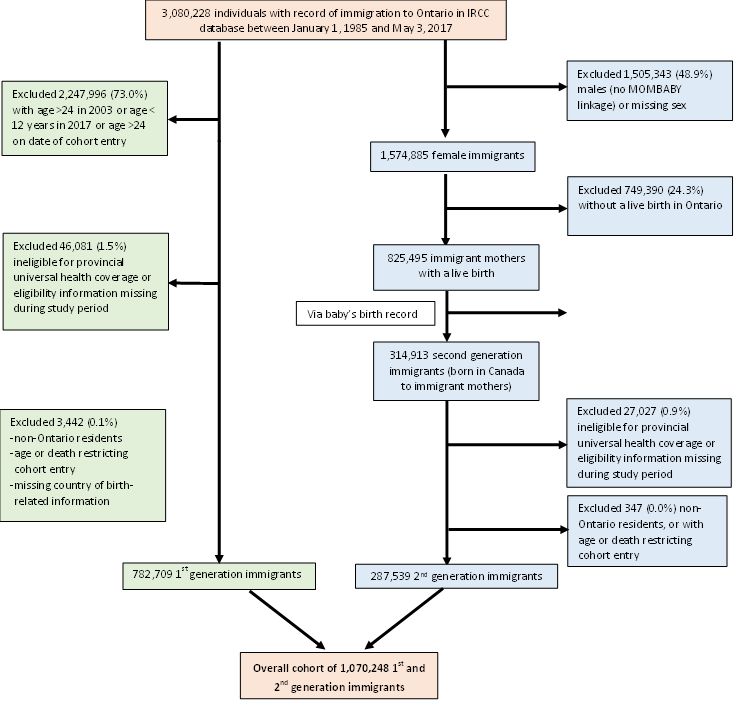

Supplement: sj-docx-1-cpa-10.1177_07067437231166840 - Supplemental material for Suicide and Self-Harm Among Immigrant Youth to Ontario, Canada From Muslim Majority Countries: A Population-Based Study [file sj-docx-1-cpa-10.1177_07067437231166840.docx]
